# The Role of Malaria Microscopy Training and Refresher Training Courses in Malaria Control Program in Iran during 2001 – 2011

**Published:** 2012

**Authors:** M Nateghpour, GhH Edrissian, A Raeisi, A Motevalli – Haghi, L Farivar, Gh Mohseni, A Rahimi-Froushani

**Affiliations:** 1 Department of Medical Parasitology and Mycology, School of Public Health, Tehran University of Medical Sciences (TUMS), Tehran, Iran; 2 National Institute of Health Research, TUMS, Tehran, Iran; 3 Center for Research of Endemic Parasites in Iran (CREPI), TUMS, Tehran, Iran; 4 Center for Disease Control and Management, Ministry of Health & Medical Education, Tehran, Iran; 5 Department of Infectious Disease, Hormozgan University of Medical Sciences, Bandar Abbas, Iran; 6 Department of Epidemiology and Biostatistics, School of Public Health, TUMS, Tehran, Iran

**Keywords:** Training courses, Malaria, Control, Iran

## Abstract

**Background:**

Malaria is still one of the most important infectious diseases in the world. The disease also is a public health problem in south and southeast of Iran. This study programmed to show the correlation between regular malaria microscopy training and refresher training courses and control of malaria in Iran.

**Methods:**

Three types of training courses were conducted in this programme including; five – day, ten – day and bimonthly training courses. Each of the training courses contained theoretical and practical sections and training impact was evaluated by practical examination and multiple-choice quizzes through pre and post tests.

**Results:**

Distribution pattern of the participants in the training and refresher training courses showed that the most participants were from Sistan & Baluchistan and Hormozgan provinces where malaria is endemic and most cases of the infection come out from these malarious areas. A total of 695 identified individuals were participated in the training courses. A significant conversely correlation was found between conducting malaria microscopy training courses and annual malaria cases in Iran.

**Conclusion:**

Conducting a suitable programme for malaria microscopy training and refresher training plays an important role in the control of malaria in endemic areas. Obviously, the decrease of malaria cases in Iran has been achieved due to some activities that malaria diagnosis training was one of them.

## Introduction

Malaria is still one of the most important infectious diseases in the world and is a life threatening in the malarious areas. According to the report of WHO, 216 million malaria cases with an estimated of 655000 deaths were officially recorded in 2010 (1). The infection continuous to be the major cause of about 30% morbidity and 15% mortality cases in some malarious areas (2). Different activities such as vector control, indoor residual spraying, using larvicidals, impregnated bed net and biological control are utilized to combat the problem. Long term experiences show that besides the administrative and vector control activities, on time active and passive case finding, prompt and accurate parasite detection and then treating by effective antimalarials can result in a considerable reduction in malaria incidence, and successful implementation of malaria control programme (3). On the other hand, accurate and on time diagnosis of malaria parasites can be achieved by trained and skilled microscopists or laboratory technicians. Therefore, training affair in the field of malaria microscopy plays an important role in proficiency of microscopists and laboratory technicians. Indeed, such wisely investment will strongly facilitate the control and elimination of malaria in malarious areas.

Malaria is a public health problem in south and southeast of Iran. The annual reports of Communicable Disease Centre (CDC) show that the number of malaria cases from 19129 cases in 2001 has been decreased to 2656 cases in 2011 (1).

This study was programmed to show the correlation between regular malaria microscopy training and refresher training courses and control of malaria in Iran.

## Material and Methods

Since long history of collaboration between Malaria Control Department, Ministry of Health and School of Public Health (Tehran University of Medical Sciences) the School of Public Health, TUMS, Iran has been designated as a research and training focal point of malaria programs including malaria microscopy. Three types of training courses were conducted in this programme including:

1. Five-day training course for refreshing knowledge of those laboratory technicians who are involved in laboratory diagnose of malaria.
2. Ten-day training course for refreshing knowledge of malaria microscopists.
3. Bimonthly training course for those eligible individuals that could be malaria microscopist in health centers.

The objectives of the training course for the first and second items were to strengthen the knowledge of participants in the field of malaria microscopy and for the third item were to train the malaria microscopy for new eligible participants. Each day of the courses covered seven training hours (theory and practical).

Training impact of the courses was evaluated by practical and multiple –choice quizzes through pre and post tests.

This study was designed as a retrospective study to consider the number of participants, their geographical distribution, and their academic grades, quality of the training and also the influence of a decade (2001 – 2011) malaria microscopy training courses on the control of malaria in Iran.

### Entry requirements

The refresher training courses were designed for microscopists and laboratory technicians and the bimonthly course was conducted for eligible high school graduated students.

### Course contents

Each of the courses was divided into two sections of training as:

1. Theoretical subjects including: Introduction to malaria microscopy, malaria parasites, life cycle and morphology of human plasmodia, morphology of blood cells, antimalarial drugs and treatment of uncomplicated malaria, drug resistance in malaria parasites and Rapid Diagnostic Tests (RDTs). The theoretical subjects were presented according to the basic malaria microscopy (4). This section in each course covered about one-third of the course duration.
2. Practical subjects including**:** blood collection, preparing thick and thin blood smears and staining them with Giemsa stain, detection of malaria parasites, differentiating between human plasmodia, artifacts, counting malaria parasites, keeping and storing the examined slides, recording the results and preparing relevant reports. The practical subjects were performed according to the standard operating procedures (SOPs) of WHO manuals (4, 5). This section of the each course covered two-third of the course duration.

## Results

**Fig. 1:**
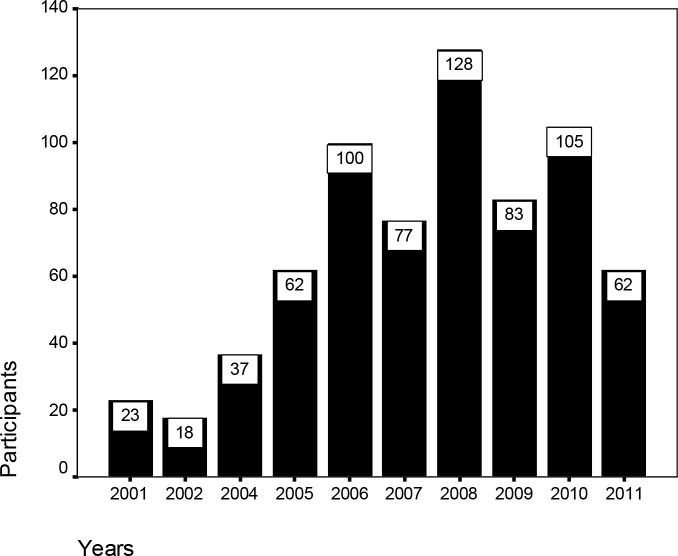
The number of individuals participated in the microscopy malaria courses during 2001- 2011

Distribution pattern of the participants in the training and refresher training courses showed that the most participants were from Sistan & Baluchistan and Hormozgan Provinces where malaria is endemic and most cases of the infection come out from these areas in Iran. Distribution pattern of the participants according to the course conduction year is shown in Fig. 1. A total of 695 identified individuals were participated in the training courses.

The Table 1 shows the number and site of the courses as well as the mean of pre and post tests in each year. The table also shows a significant difference between the grades of pre & post tests (*P* <0.001). According to this table most of the courses were conducted at the Bandar-Abbas Health Research Station in Hormozgan Province.

**Table 1:** The number of participants, courses, site of the courses and mean of pre & Post tests in 2001 – 2011*

Years	Courses(no.)	Participants(no.)	Site of courses	Pre tests mean	Post tests mean
2001	1	23	Tehran**	11.57	17.02
2002	1	18	Bandar- Abbas ***	11	17.30
2004	1	18	Bandar- Abbas	10.36	18.58
	1	19	Bandar- Abbas	9.19	18.67
2005	1	20	Tehran	7.28	18.41
	1	21	Bandar- Abbas	11.88	19.45
	1	21	Bandar- Abbas	12.36	18.72
2006	1	20	Bandar- Abbas	9.20	18.42
	1	23	Tehran	10.26	19.18
	1	22	Bandar- Abbas	11.41	19.10
	1	21	Tehran	9.26	19.35
	1	14	Qrumiyeh	9.95	17.54
2007	1	23	Bandar- Abbas	9.94	19.48
	1	19	Tehran	10.37	19.50
	1	21	Bandar- Abbas	9.79	19.23
	1	14	Tehran	8.75	18.94
2008	1	28	Bandar- Abbas	11.51	19.55
	1	10	Tehran	10.70	19.87
	1	22	Bandar- Abbas	11.28	18.23
	1	23	Bandar- Abbas	10.90	18.63
	1	24	Iranshahr	11.72	18.14
	1	21	Tehran	10.05	18.59
2009	1	21	Bandar- Abbas	10.61	18.46
	1	20	Bandar- Abbas	7.23	19.26
	1	24	Bandar- Abbas	11.90	18.97
	1	18	Tehran	11.35	19.31
2010	1	23	Bandar- Abbas	12.74	18.17
	1	21	Tehran	10.59	19.46
	1	20	Tehran	11.13	18.89
	1	21	Bandar- Abbas	9.62	18.80
	1	20	Tehran	12.01	19.46
2011	1	17	Tehran	9	1849
	1	19	Tehran	10.3	19.17
	1	14	Bandar- Abbas	10.02	19.16
	1	12	Tehran	12.82	19.32
Total	35	695	-	10.57	18.81

* There did not conduct any course in 2003 due to some planning problems.

** School of Public Health

*** Bandar – Abbas Health Research Station

**Fig. 2:**
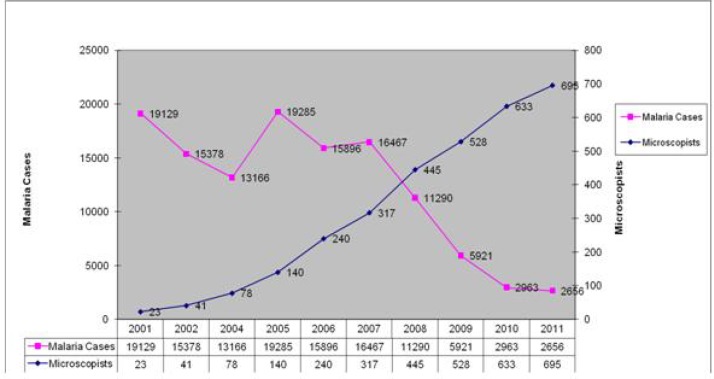
Comparison between trend of malaria cases and number of trained malaria microscopists during 2001-2011

Correlation between conducting malaria microscopy training courses and annual malaria cases in Iran is illustrated in Fig. 2.

## Discussion

In addition to well adopted administrative and vector control activities, prompt case finding in malaria infection and accurate treatment of the disease plays an important role in control and elimination of malaria. On the other hand, on time treatment of malaria infection depends on the exact and swift diagnosis of malaria parasites.

Although clinical signs particularly in malarious areas can guide the physicians to suspect malaria infection, it is not sufficiently sensitive and specific. Due to avoiding presumptive treatment for malaria in cases come out with fever, the current WHO recommendation emphasizes on the systematic testing of all fever cases (6). Detection of malaria parasites and differentiating between them is crucial for accurate treatment of the infections.

Although some other methods such as PCR, Quantitative Buffy Coat (QBC), Indirect Fluorescent Antibody (IFA) and light microscopy can be used for diagnosis of malaria parasites, among them light microscopy is the conventional and method of choice for malaria case detection of malaria parasites in the most of malarious areas.

In the light microscopical method prompt and exact diagnosis also depends on two principles; well trained and skillful microscopists and good materials and equipments. The latter is usually available in the markets, but rearing the competent microscopists takes time and needs adequate expenditures. Such aim can be achieved with designing a reasonable programme. The results of this study show that employing the trained malaria microscopists in their right place will lead to the desirable results (Fig. 2). Finding from some studies in Africa counted a number of facts for poor performance of routine microscopy in health facilities that insufficient training was one of the leading rationale for the shortcomings (2, 7). A 3-day refresher training programme conducted in Uganda could significantly improve the knowledge of health facilities staff in the field of practical malaria microscopy (8). Kahama-Maro et al. showed that 53% of blood films were diagnosed as positive by routine laboratories, whereas only 2% of the films were found positive by expert microscopy during the re-checking process. They concluded that such poor implementation of routine microscopy is due to several factors including lack of competency in laboratory technicians (9). Our long term experiences show that training and refresher training courses of malaria can significantly affect on the competency of microscopists and laboratory technicians. According to our knowledge most of the trained and refresher trained individuals included in this study were engaged with malaria affairs particularly in the field of malaria microscopy. Theoretical and practical evaluation in each course showed considerable improvement in most of the participants. The evaluation indicated that participants from non-endemic districts need more retraining than those who were from malaria endemic districts.

## Conclusion

Preparing a suitable programme for training and refresher training courses in the field of malaria microscopy can have a considerable impact on the knowledge and competency of the microscopists and laboratory technicians as well as plays an important role on control of malaria in endemic areas.

Obviously, the decrease of malaria cases in Iran has been achieved due to some activities that malaria diagnosis training was one of them.
